# Role of ubiquitin specific proteases in the immune microenvironment of prostate cancer: A new direction

**DOI:** 10.3389/fonc.2022.955718

**Published:** 2022-07-18

**Authors:** Jinhui Guo, Jie Zhao, Litao Sun, Chen Yang

**Affiliations:** ^1^ Cancer Center, Institute of clinical medicine, Zhejiang Provincial People’s Hospital, Affiliated People’s Hospital, Hangzhou Medical College, Hangzhou, China; ^2^ Qingdao Medical College, Qingdao University, Qingdao, China; ^3^ College of Biotechnology and Bioengineering, Zhejiang University of Technology, Hangzhou, China; ^4^ Cancer Center, Department of Ultrasound, Zhejiang Provincial People’s Hospital, Affiliated People’s Hospital, Hangzhou Medical College, Hangzhou, China

**Keywords:** ubiquitylation, deubiquitination, prostate cancer, USPs, tumor microenvironment, immune evasion

## Abstract

Regulation of ubiquitination is associated with multiple processes of tumorigenesis and development, including regulation of the tumor immune microenvironment. Deubiquitinating enzymes (DUBs) can remove ubiquitin chains from substrates, thereby stabilizing target proteins and altering and remodeling biological processes. During tumorigenesis, deubiquitination-altered biological processes are closely related to tumor metabolism, stemness, and the immune microenvironment. Recently, tumor microenvironment (TME) modulation strategies have attracted considerable attention in cancer immunotherapy. Targeting immunosuppressive mechanisms in the TME has revolutionized cancer therapy. Prostate cancer (PC) is one of the most common cancers and the second most common cause of cancer-related death in men worldwide. While immune checkpoint inhibition has produced meaningful therapeutic effects in many cancer types, clinical trials of anti-CTLA4 or anti-PD1 have not shown a clear advantage in PC patients. TME affects PC progression and also enables tumor cell immune evasion by activating the PD-1/PD-L1 axis. Over the past few decades, an increasing number of studies have demonstrated that deubiquitination in PC immune microenvironment may modulate the host immune system’s response to the tumor. As the largest and most diverse group of DUBs, ubiquitin-specific proteases (USPs) play an important role in regulating T cell development and function. According to current studies, USPs exhibit a high expression signature in PC and may promote tumorigenesis. Elevated expression of USPs often indicates poor tumor prognosis, suggesting that USPs are expected to develop as the markers of tumor prognosis and even potential drug targets for anti-tumor therapy. Herein, we first summarized recent advances of USPs in PC and focused on the relationship between USPs and immunity. Additionally, we clarified the resistance mechanisms of USPs to targeted drugs in PC. Finally, we reviewed the major achievement of targeting USPs in cancers.

## Introduction

Prostate cancer (PC) is one of the leading causes of morbidity and mortality in men worldwide (1). Radical surgical resection combined with androgen deprivation therapy (ADT) can be selected for the treatment of localized disease (2). Even though local treatment reduces mortality in PC patients, 20-40% of men experience recurrence (3). Although the initial effect of ADT is significant, this subset of patients will eventually progress to castration-resistant PC (CRPC) (4). Once PC spreads, the survival rate drops significantly to around 30% (5). Despite the success of ADT, chemotherapeutics, and radiopharmaceuticals in PC, none of these therapies cures advanced PC (6–8). As a novel treatment, immunotherapy has achieved remarkable success in solid tumors, such as melanoma, but has shown limited therapeutic benefits in PC. The insensitivity of PC to immunotherapy (such as checkpoint inhibitors) may reflect the immunosuppressive nature of the tumor microenvironment (TME) in PC. Cells within the TME express and secrete molecules, including programmed death-ligand 1 (PD-L1), transforming growth factor-beta (TGF-β), and vascular endothelial growth factor (VEGF) to mediate immunosuppression. Additionally, immune tolerance plays a key role in the occurrence and development of prostate tumors (9, 10).

Ubiquitination is involved in nearly all cellular processes, including protein activation/inactivation, DNA repair, gene regulation, and signal transduction (11, 12). In addition to these broad roles, ubiquitination was closely associated with the regulation of immune responses, as well as immune tolerance (13). Ubiquitination regulates T cell development, activation, and differentiation, thereby maintaining adaptive immune responses and immune tolerance to self-tissues (13). Many proteins in the T cell receptor (TCR) signaling pathway are regulated by the ubiquitin-proteasome system (UPS), which is critical for T-cell activation (14).

**Figure 1:**
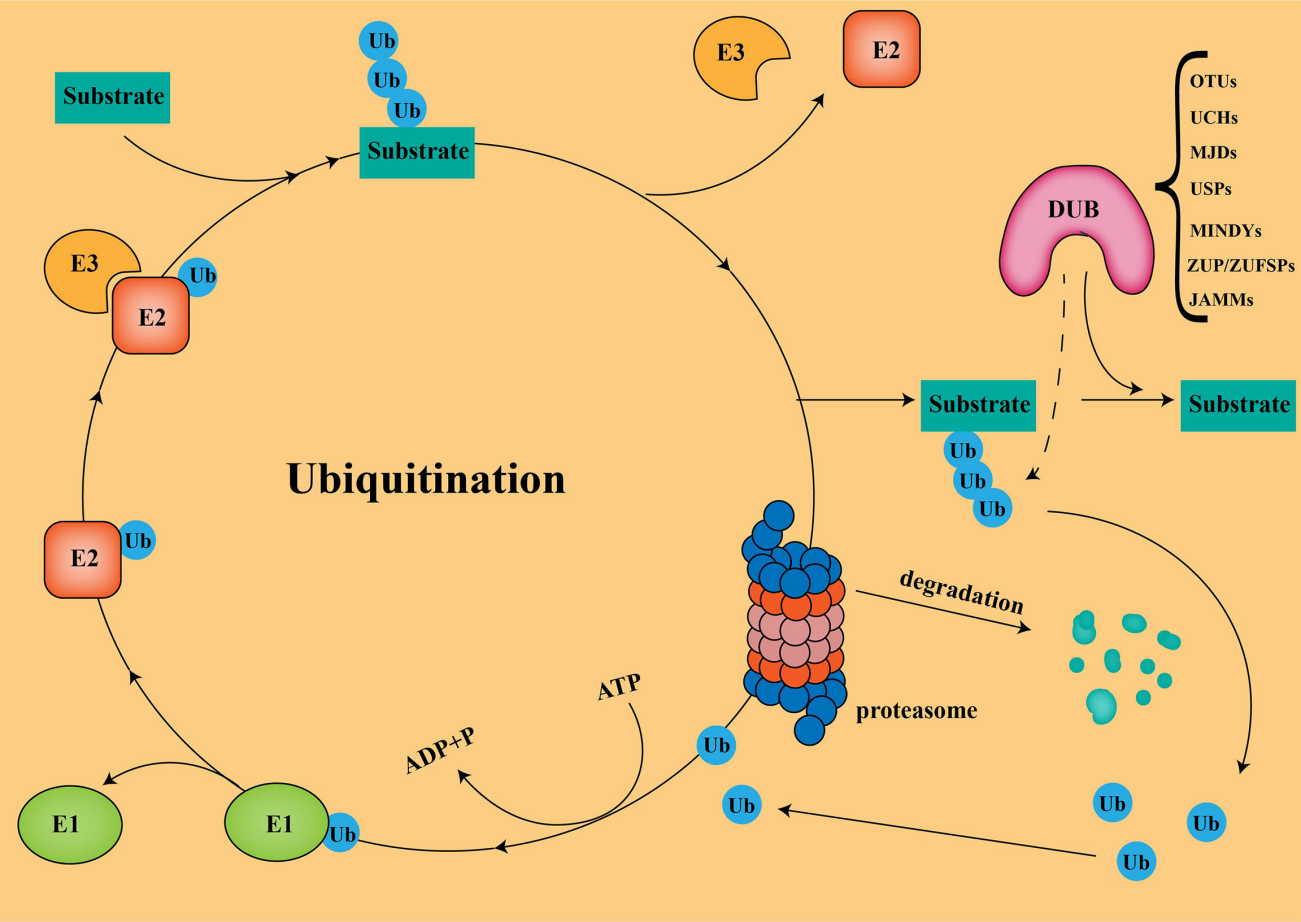
Ubiquitination and Deubiquitination The substrate proteins are covalently attached to ubiquitin *via* isopeptide bonds catalyzed by an E1-E2-E3ligase cascade, followed by proteasomal degradation.

In the UPS, the substrate proteins are covalently attached to ubiquitin *via* isopeptide bonds catalyzed by an E1-E2-E3 ligase cascade, followed by proteasomal degradation (**Figure 1**) (15). It should be noted that not all ubiquitination modifications lead to protein degradation. Some ubiquitination do not degrade proteins, but alters protein activity, thereby mediating biological effects, such as gene regulation and DNA damage repair (12). Ubiquitin molecules are linked to target proteins as mono- or poly-ubiquitin. Generally, polyubiquitination marks signals for protein degradation by cellular proteasomes, while monoubiquitination marks can act as non-degradative modifications. Ubiquitins are mainly connected by lysine (K6, K11, K27, K29, K33, K48, and K63) and methionine (M1) residues. The K48 and K63 chains are the most studied ubiquitin chain linkages that guide the expression of substrate proteins. The K48 ubiquitin chain has been shown to play an important role in ATP-dependent proteasomal degradation (16), while the K63 ubiquitin chain is mainly involved in the modification of protein location and function (17).

Deubiquitinases (DUBs) regulate a variety of cellular functions by removing ubiquitin chains from substrates. Currently, more than 100 DUBs have been found in humans, and are divided into seven different families according to their structure and function (18): ubiquitin-specific proteases (USPs), ovarian tumor proteases(OTUs), ubiquitin C-terminal hydrolases (UCHs), Jab1/Mov34/MPN+ proteases (JAMMs), Machado-Joseph disease protein proteases (MJDs), the motif interacting with ubiquitins(MIUs)-containing novel DUB family members (MINDYs), and Zinc Finger ubiquitin-specific proteases (ZUP/ZUFSPs). USPs is the largest and most diverse group of DUBs, accounting for about 60%, ranging between 50-300 kDa in size (19). To date, finding effective ways to enhance tumor immunotherapy in PC has been a great challenge. Turning “cold” tumors into “hot” tumors by modulating USPs to influence the immune response of the TME could effectively improve the efficacy of checkpoint inhibitors. Combining small-molecule inhibitors of USPs with checkpoint inhibitors in PC will be a promising therapeutic strategy.

## USPs and PC

### Introduction of USPs

USPs belong to the family of cysteine proteases whose enzymatic activity is located in the thiol group of the central cysteine (20). The catalytic sites all contain a cysteine residue with nearby histidine and asparagine/aspartate residues that facilitate nucleophilic attack by the cysteine (21). USPs promote the occurrence and development of PC by participating in multiple signaling pathways, such as androgen receptor (AR) accumulation, TGF-β pathway, and p53 pathway (22). Additionally, deubiquitination of USP can also regulate the AKT phosphorylation and fatty acid synthase pathways in PC (22, 23).

AR is the most common cause of signaling pathways in PC and may contribute to the emergence of CRPC (24). Activation of AR inhibits proteasomal degradation of MYC, leading to PC cell invasion (25). In primary PC, MYC is commonly amplified and overexpressed in 37% of metastatic focus (26, 27). MYC was regarded as the key driver of CRPC pathogenesis, and its amplification usually indicated poor outcomes (28). Stability of MYC is precisely regulated by UPS, which further regulates the growth of PC cells. Multiple USPs (USP2a, USP16, and USP22) are known to regulate MYC stability (29–31). USP22 is a functional mediator necessary for MYC to exert oncogenicity, thereby increasing the stability and tumorigenic activity of MYC in PC cells (31).

Damage to DNA triggers corresponding cellular responses, ranging from arresting the cell cycle to activating specific DNA repair mechanisms that vary, depending on the type of damage (32). Unrepaired DNA damage disrupts genome integrity and contributes to the pathogenesis of a range of human diseases, including cancer and premature aging (33). Additionally, DNA damage-induced senescence is associated with a pro-inflammatory secretory phenotype that remodels the tumor immune microenvironment (34). This study found that some USP families are also involved in the DNA damage repair pathway. Both USP26 and USP37 participate in homologous recombination by regulating rap80, and then repair DNA double-strand break (DSB) (35). Additionally, USP14 regulates DNA damage repair by targeting RNF168-dependent ubiquitination (36). During nucleotide excision repair, the nucleotide excision repair protein represented by XPC could repair the damaged DNA by ubiquitination binding (37). The USP22 could significantly protect XPC from deubiquitination; thus, promoting the survival of damaged DNA (38). Additionally, USP7 and USP11 can also regulate nucleotide excision repair through deubiquitinating XPC (39, 40).

### USPs maintain AR stability

AR, a steroid receptor transcription factor for testosterone and dihydrotestosterone, is a central driver of PC development. Androgens act as ligands that bind to AR, and the activated AR binds to the DNA sequences of downstream genes, which initiates the expression of a series of genes that promote PC progression. Prostate-specific antigen is the most well-characterized AR target for monitoring PC development. Due to the central role of AR signaling in PC progression, ADT therapy has been the mainstay of treatment for patients with locally advanced PC. Reactivation of AR signaling can still be detected in CRPC cells despite multiple therapeutic options for inhibiting AR signaling (41). Amplification and mutation of the AR gene are also associated with the progression of CRPC, ultimately rendering it incurable.

Most of the previous studies have focused on the regulation of AR synthesis in PC, while the regulation of AR post-translational modification and degradation is easily overlooked. Several USPs (USP7, USP10, USP12, USP14, USP22, and USP26) have been reported to regulate AR signaling pathways, thereby affecting AR stability in the prostate. USPs (USP7, USP12, USP14, and USP22) can directly deubiquitinate AR and promote AR transcription (31, 42–44). USPs (USP12, USP14, and USP26) can also protect AR through reduced ubiquitination and degradation by indirectly reducing mouse double minute 2 (MDM2, a negative regulator of the tumor suppressor p53) protein levels (44–46). Furthermore, USP7 and USP10 can indirectly stabilize AR through histone H2A deubiquitination (42, 47).

### Resistance of enzalutamide

Enzalutamide is a next-generation AR pathway inhibitor that binds to the ligand-binding domain of AR and disrupts the interaction between AR and androgen. Enzalutamide was initially effective in men with hormone-sensitive PC, but in most cases, resistance to the therapy tends to develop over time. Overexpression of some USPs in PC inhibits the enzalutamide effect and confers resistance to ADT therapy. Overexpression of USP22 enhanced AR protein accumulation, which in turn activated downstream target genes regulated by AR and MYC. Such USP22-mediated activation can bypass androgens or AR antagonists (enzalutamide) to induce castration resistance in PC (48). Androgen receptor splice variant 7 (AR-V7), a ligand-independent activating variant of AR, is thought to be an inducer of CRPC. Targeted AR therapy is limited in CRPC due to lack of the ligand-binding domain of AR-V7. Among the AR-Vs, AR-V7 is the most abundant variant that has the highest detection frequency in PC. It should be noted that AR-V7 is the only endogenous variant detected at the protein level and can show functional activity in the absence of androgens. Protein analysis showed that USP22 depletion significantly reduced the half-life of AR-V7. Conversely, overexpression of USP22 slowed down AR-V7 degradation to some extent, partially restoring the viability of CRPC cells (48).

The kinesin family member 15 (KIF15) promotes enzalutamide resistance by enhancing AR signaling in PC cells. The KIF15 directly binds to the N-terminus of AR/AR-V7 and prevents AR/AR-V7 protein degradation by increasing USP14 binding to AR/AR-V7 (49). KDM4A demethylates the promoters or enhancers of certain AR target genes and acts as an AR co-activator. USP1 deubiquitinates and stabilizes KDM4A, thereby promoting the binding of AR to the c-MYC gene enhancer. Furthermore, inhibition of USP1 reduced PC proliferation and promoted resistance to enzalutamide in a KDM4A-dependent manner (50).

## TME and USPs

### Background of TME

The TME consists of tumor cells, immune cells, fibroblasts, endothelial and inflammatory cells, lymphocytes, and extracellular matrix (ECM) (51). Infiltration of immune cells (CD4+ and CD8+ T cells, dendritic cells, and natural killer cells) into tumors is associated with improved prognosis in cancer patients. Impaired cellular immunity and immunosuppressive TME may lead to PC becoming a “cold” tumor (52). In advanced PC, the function of natural killer and T cells is impaired in the TME, and myeloid suppressor cells and regulatory T cells (Tregs) are increased.

Cancer-associated fibroblasts (CAFs), a heterogeneous population of mesenchymal cells, are major players in the tumor immunosuppressive system. VEGF is a key factor secreted by CAFs to stimulate new blood vessel formation. By targeting the VEGF/VEGF receptor signaling pathway, the proliferation of tumor endothelial cells (TECs) can be inhibited, thereby controlling neovascularization in the TME. The CAFs can also build microenvironmental structures by synthesizing large amounts of ECM in the TME with important implications for maintaining stemness, regulating tumor metabolism, and therapeutic resistance. More and more researchers are paying attention to the immunosuppressive effects of CAFs through their interactions with components of the immune TME, especially immune cells (53, 54). In PC, M2 macrophages stimulate CAFs development by triggering neovascularization, both of which synergize with tumor development (55). The upregulated USP24 in M2 tumor-associated macrophages (TAMs) could promote the malignant development of cancer by increasing IL-6 expression (56). Additionally, USP22 has an important function in repairing DSBs that occur during B cell development (57).

### Myeloid-derived suppressor cells

MDSCs are a heterogeneous population of immature myeloid cells that suppress T and NK cellular activity, and they can also confer resistance of tumor cells to immunotherapy. Clinical trials have found a correlation between MDSCs abundance and poor response to checkpoint inhibitor intervention (58). The recruitment of immune cells involved C-X-C motif chemokine ligand 5 (CXCR2), which promotes angiogenesis and tumor growth (59). CXCR2 plays a role in tumor progression by promoting the migration of MDSCs into the TME (60). MDSCs are enriched in prostate tumors in a CXCR2-dependent manner after surgical castration. Mast cells are innate immune cells and the number of infiltrating human prostate cancer correlates with prognosis (61). Mast cells can interact with MDSCs *via* CD40, further enhancing immunosuppression and directly impairing CD8+ T cell function (62). The extent of T-cell infiltration in prostate tumors is inversely related to the frequency of MDSCs, showing a strong synergistic response when MDSC-targeted therapy is combined with checkpoint inhibitors (63). Tyrosine kinase inhibitors can enhance the effect of immune checkpoint inhibitors by downregulating various cytokines that promote immunosuppression in MDSCs. The MDSCs may be a useful therapeutic target in the immune microenvironment of PC. Studies have found that USP22 deletion may lead to a significant reduction of MDSCs in the TME and promote the infiltration of T cells and NK cells while the expression of USP22 confers tumor resistance to immunotherapy (64).

### Tregs and PD1/PDL1

Tregs are a unique class of immunosuppressive CD4+ T cells that primarily suppress the immune system by expressing the master transcription factor forkhead box protein 3 (FOXP3). Tregs penetrate the TME and suppress antitumor immune responses, and the ratio of Tregs to T cells reveals the effect of immunotherapy (65, 66). Tregs can inhibit antigen-presenting cells (APCs), thereby producing immunosuppressive factors, leading to the development of immunosuppressive TME.

**Figure 2:**
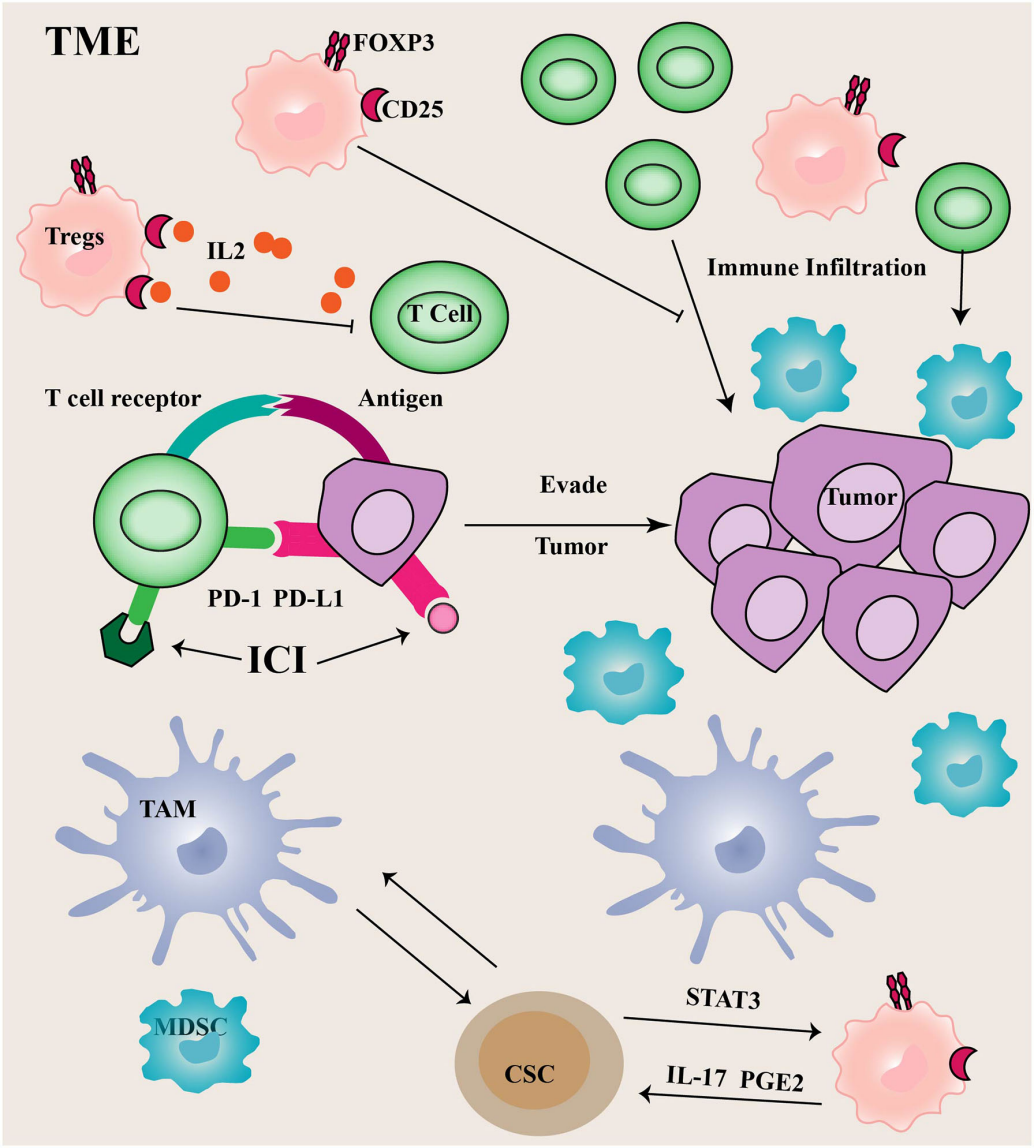
Immune cells in TME Tumor cells often utilize upregulated PD-1 ligands to induce T cell exhaustion, thereby promoting tumor immune escape.

FOXP3 is a hallmark transcription factor that determines and maintains the functional program of Tregs (67). It inhibits interleukin-2 (IL-2) transcription and induces CD25 expression (68). CD25 is a high-affinity receptor for IL-2, IL-2 is an essential cytokine for the survival of Tregs and effector T cells, thus, CD25 expression in Tregs can compete for more IL-2 binding in the TME than effector T cells. Therefore, Tregs accumulate more in the TME than effector T cells (**Figure 2**) (69). FOXP3 protein expression can be regulated by polyubiquitination-mediated proteasomal degradation. Expression of USP7 is up-regulated and active in Tregs cells, associated with FOXP3 in the nucleus. Ectopic expression of USP7 decreased FOXP3 polyubiquitination and increased FOXP3 expression. USP7 knockdown treatment reduced the expression of endogenous FOXP3 protein, and decreased Tregs cell-mediated inhibition *in vitro* (70).

PD-1 limits immune responses primarily by inhibiting intracellular signaling in effector T cells. Compared to CTLA4, the PD-1/PD-L1 (the ligand of PD-1) axis is more critical for the continued activation and proliferation of differentiated effector cells. The binding of PD-1 to PD-L1 can mediate T cell apoptosis or induce T cell dysfunction, commonly referred to as T cell exhaustion. Additionally, PD-L1 plays an important role in regulating immune responses (71). The PD-L1 is normally expressed on APCs and can control Tregs differentiation and inhibit their activity. Tumor cells and other TME components, such as infiltrating myeloid and dendritic cells often utilize upregulated PD-1 ligands to induce T cell exhaustion, thereby promoting tumor immune escape. Depletion of USP22 promotes T cell-mediated cell killing. Moreover, USP22 could regulate PD-L1 levels through two pathways (72). On the one hand, USP22 can directly regulate PD-L1 degradation through deubiquitination. On the other hand, USP22 regulates the expression of PD-L1 through the USP22-CSN5-PD-L1 axis.

## Cancer stem cells and TME

CSCs are a subpopulation of undifferentiated cancer cells within a tumor with the ability to self-renew and differentiate into multilineages. The expression of several stem cell surface markers (CD44, CD133, OCT4, SOX2, and NANOG) has been associated with the promotion of treatment resistance and cancer progression. The TME is characterized by chronic inflammation that activates and modulates CSCs by stimulating cell proliferation (73). Recent studies have revealed a close connection between immune cells in CSCs and TME (74). TAMs provide key signals to promote CSCs survival, self-renewal, maintenance, and migration capabilities, and the CSCs provide tumor-promoting signals to TAMs in turn, further enhancing tumorigenesis. The CSCs make dendritic cells tolerant and impede the aggregation of dendritic cells in the TME. CSCs may also overcome immune surveillance by inhibiting T cell proliferation and effector function (75, 76). CSCs induce Tregs infiltration through STAT3 signaling in the TME, while Tregs regulate CSCs proliferation and expansion through IL-17 and PGE2 (77). In conclusion, CSC-targeted immunotherapy has the potential to become a new type of immunotherapy for cancer.

**Figure 3:**
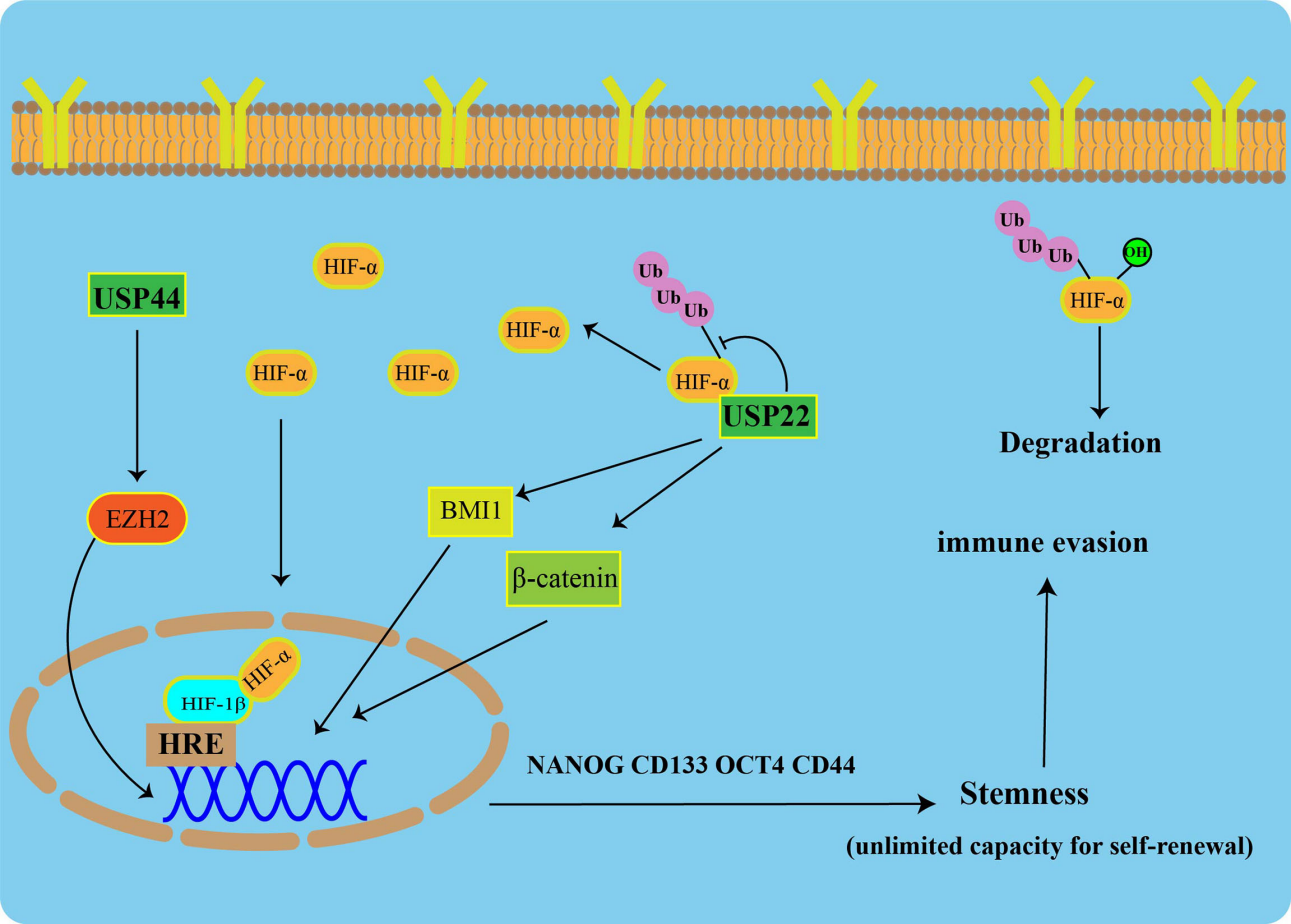
USPs-mediated tumor stemness and hypoxic microenvironment Under normoxic conditions, HIF-1α is degraded *via* the ubiquitin-proteasome pathway. USP22 enhances the stability and transcriptional activity of HIF-1α under hypoxia through deubiquitination and induces upregulation of HIF-1α downstream genes. USP22 maintains cancer stemness by regulating BMI1 and β-catenin pathways. Furthermore, USP44 promotes prostate cancer stemness by deubiquitinating EZH2.

To maintain CSC pluripotency, post-translational modifications (such as ubiquitination) are tightly regulated. USP22 is increased during progression from early-stage PC to CRPC, and it has a strong prognostic value in PC (78). In multiple tumors, USP22 has been described as a CSC marker that promotes CSC formation and stemness maintenance (79). USP22 affects the self-renewal of CSCs in cancer by regulating BMI1 protein expression (80). Additionally, USP22 promotes CSC maintenance through the Wnt/β-catenin pathway (81). Apart from USP22, USP44 has been shown to be upregulated in CSCs of breast cancer and promote tumor angiogenesis. In PC, knockdown of USP44 suppressed CSC properties and reduced the tumorigenicity of the PC. The expression of some pluripotent stem cell markers (OCT4, NANOG, and CD133) was reduced in USP44 knockdown cells. Specifically, USP44 promotes PC stemness by deubiquitinating EZH2 (a histone-modifying enzyme). The introduction of the ectopic EZH2 rescues the suppression of tumor activity after the USP44 knockout (**Figure 3**) (82).

### Hypoxic microenvironment

The characteristics of vascular tortuosity and rapid tumor cell growth in TME usually induce hypoxia and recruit immunosuppressive cells, including macrophages, Tregs, and MDSCs. These immunosuppressive cells contribute to immunosuppression in the TME by secreting immunosuppressive factors, such as VEGF and TGF-β. Additionally, such a hypoxic microenvironment may lead to a polarization state transition of microphages from M1 to M2 based on their plastic properties.

Hypoxia is difficult to avoid in the process of prostate treatment. ADT therapy induces a hypoxic microenvironment in PC and triggers autocrine TGF-β signaling and differentiation of CAFs into myofibroblasts (83). Unfortunately, in a hypoxic environment, tumors can resist T cell infiltration even in the context of checkpoint inhibitors (CTLA-4 and PD-1 blocking). Studies in mouse tumor models of PC show that hypoxic regions of tumors represent centers of immunotherapy resistance, promoting the transformation of immature myeloid cells into highly suppressive myeloid-derived suppressor cells (84). Hypoxia can alter vasculature in the TME through direct (Hypoxia-inducible factor 1α-mediated upregulation of VEGF) and indirect effects (accumulation of cells leading to abnormal angiogenesis). The resulting vasculature expresses too few adhesion molecules necessary to support T cell extravasation, which can actively induce T cell apoptosis through the involvement of the Fas receptor. Those T cells capable of entering hypoxic tumors face a metabolically, highly inhibited immune environment (dense expression of PD-L and high concentrations of TGF-β). Additionally, the MDSCs are concentrated in the hypoxic regions of these tumors and they form an effective barrier to tumor immunity. Hypoxia itself inhibits T effector cell differentiation and interferon-γ production (85).

A heterodimeric transcription factor, Hypoxia-inducible factor 1(HIF-1), consist of an α subunit expressed in an oxygen-dependent manner and a constitutively expressed β subunit. Under normal toxic conditions, HIF-1α is degraded *via* the UPS. USP22 enhances the stability and transcriptional activity of HIF-1α under hypoxia through deubiquitination, and it induces upregulation of HIF-1α downstream genes (**Figure 3**) (86).

## Important signaling pathways in PC TME

### P53

P53 (also known as TP53) is a tumor suppressor, and P53 degradation or gene mutation is tightly involved in cancer formation (87). P53 is a major regulatory transcription factor capable of regulating a variety of biological processes, such as cell cycle arrest, apoptosis, senescence, and repair of DNA damage (88). In addition to the known oncogenic role of P53, P53 plays an important role in immune responses and inflammation. P53 affects immunity and inflammation by regulating Toll-like receptors (TLRs) (89). TLRs are an important class of protein molecules involved in nonspecific immunity and are also a bridge between nonspecific and specific immunities. Changes in TLRs expression are associated with autoimmune diseases. There is also an important link between P53 and immune checkpoints. Cancer cells suppress immune responses and evade immune surveillance by upregulating PD-1 and its ligand PD-L1 in a P53-dependent manner. Another immune checkpoint regulator, DD1α, is also a direct transcriptional target of P53 (90). Many P53-regulated microRNAs (miRNAs) are also implicated in immunity. For example, miR34 binds directly to the 3′ untranslated region of the gene encoding PD-L1, suggesting that P53 may regulate tumor immune responses through miR-34 regulation of PD-L1 expression (91).

Mutant P53 (MTP53) protein is a tumor-specific neoantigen that is immunogenic and can mediate the immune escape of cancer cells. MTP53 makes tumors immunologically “cold” by inhibiting the STING-TBK1-IRF3 pathway, thereby allowing cancer cells expressing the “MTP53” antigen to evade immune detection (92). Importantly, disruption of the MTP53/TBK1 complex can switch the TME from “cold” to “hot” and allow the immune system to limit tumor growth. Other (TBK1-independent) mutant P53 activity may also contribute to the TME regulation. P53 also regulates the polarization of CD4+ T cells by enhancing the transcription of Foxp3, the master regulator of Tregs, and it is therefore predicted that loss of this role of p53 might enhance antitumor immunity. Targeting the P53-MDM2 interaction enhances MDM2 in T cells, thereby stabilizing STAT5 and enhancing T-cell-mediated antitumor immunity (93).

Some USPs can modulate p53 through deubiquitination or indirectly affect p53 through other signaling pathways. USP10 can interact with G3BP2 to block P53 signaling, leading to a poor prognosis in PC (94). USP12 and USP2a directly deubiquitinate and stabilize MDM2, thereby controlling the level of P53 gene in PC (45, 95). Downregulation of USP7 can increase the level of P53 *via* promoting MDM2 degradation (96). Caspase 8, a member of the cysteine protease family, is a key driver of apoptotic cell death. In cells with caspase 8 depletion, USP28 stabilizes p53 by deubiquitination to induce apoptosis of PC cells. However, in the presence of nuclear caspase 8, USP28 is cleaved and inactivated, resulting in the loss of p53 protein (97). Therefore, the dependence of caspase 8 should be considered, while developing drugs against USP28 inhibitors in the future.

### TGF-β

TGF-β is a pleiotropic cytokine with a complex role in cancer progression (98). In the TME, cancer cells can exploit the pleiotropy of TGF-β signaling and its downstream mediators to create an immunosuppressive environment to evade antitumor immunity. TGF-β mediates endothelial-mesenchymal transition through SNAIL/Slug expression in TECs to support neovascularization and accumulation of myofibroblasts and CAFs in the TME (99). Moreover, TGF-β can drive immune dysfunction in the TME by inducing regulatory T cells and suppressing CD8+ and TH1 cells (100). TGF-β inhibits interferon-γ expression, restricts TH1 cell differentiation, attenuates CD8+ activation and effector T cell killing, and inhibits central memory T cell development. TGF-β induces the differentiation of CD4+ T cells in the TME into Tregs and has a major impact on the prognosis of patients with this functional tumor.

Studies have shown that USPs (USP2a, USP4, USP9X, USP15, and USP26) are involved in the regulation of TGF-β signaling pathways in various cancers (17). For example, USP9X can control the monoubiquitination of SMAD4 to regulate TGF-β-mediated cancer metastasis (101).

## Small molecule inhibitors of USPs

Based on the accumulated evidence that indicated the potential of USP to promote cancer, targeting USP therapy strategy has attracted extensive attention. In the past few years, great breakthroughs have been made in the screening and development of small-molecule USPs inhibitors. USP14 is the most studied member of the USPs family. Herein, we use USP14 as a representative to describe small molecule inhibitors. USP14 contains a total of 494 amino acids with a UBL domain at its N-terminus and a catalytic USP domain at its C-terminus (102). The UBL domain is an important regulator of proteasome activity, while the C-terminal USP domain is responsible for its deubiquitinase activity (103). Structural studies show that two surface loops BL1 and BL2 on free USP14 prevent active site binding to the C-terminus of ubiquitin. The binding of USP14 to the 26S proteasome in an autoinhibited state activates its deubiquitination function.

In 2010, Finley et al. was the first to report a highly selective inhibitor of proteasome-bound USP14 named IU1 (104). As a specific inhibitor of USP14, IU1 has been used in cell-based studies. Because DUBs are highly conserved, previous work has mainly focused on covalent inhibitors, which are compounds that form covalent bonds with active site cysteines. However, these compounds generally have poor selectivity for the DUBs family and cannot be used clinically (105). The IU1 prevents the C-terminus of ubiquitin from binding to USP14 by binding to the thumb-palm cleft region of the catalytic domain of USP14 (106). The results of this work suggest that allosteric modulation *via* steric retardation may be a viable approach for the discovery of USP inhibitors. The IU2 series is another class of USP14 inhibitors belonging to the tricyclic thiophene derivatives. The inhibitory effect of IU2 may block the entry of ubiquitinated substrates by blocking the ubiquitin-binding pocket.

However, the low inhibitory efficiency of IU1 hinders the development of drugs targeting USP14. Additionally, no selective inhibitor against phosphorylated USP14 has been found to date. The USP14 not only affects tumor progression but also plays a key role in immune and inflammatory responses. Inhibition of USP14 activity blocks IL-1β release and caspase-1 activation, showing its therapeutic potential in autoinflammatory diseases (107). Inhibition of USP14 may have broad biological effects leading to unpredictable toxicity. For example, loss of USP14 alters synaptic activity, leading to neuronal dysfunction (108). Therefore, it is important to develop a specific inhibitor that only targets the interaction of USP14 with some of its substrates.

USP22 exerts tumor-promoting effects in multiple tumor types and suppresses anti-tumor immunity by stabilizing PD-L1 in tumors (64). Increasing evidence showed that prescribing USP22 inhibitors is desirable. The USP22 has been studied in cancer for more than 15 years, but inhibitors of USP22 have not been reported until recent studies. Morgan et al. screened these cyclic peptides in a high-throughput manner based on RaPID, a combinatorial library system containing 1012 structurally unique cyclic peptides, and finally assessed their ability to inhibit DUB activity *in vitro* based on binding affinity, to develop effective and highly specific DUB inhibitor (109). The identification of ubiquitin variants targeting specific USPs by Sidhu laboratory provides new directions for designing small molecule inhibitors of USP22. Tang et al. designed a new library of combinatorial ubiquitin variants (UbVs) with high affinity and specificity for their cognate target domains found in ubiquitin specific protease (DUSPs). UbV probes can serve as potential targets for inhibition of USPs. This suppression mechanism can be extended to other USPs containing DUSPs (110).

## Conclusion and future perspectives

Sipuleucel-T, an autologous cellular immunotherapy made from APCs, is the first FDA approved cancer vaccine for the treatment of metastatic CRPC (mCRPC), proving the prospect of immunotherapy in PC. Low tumor T-cell infiltration is one of the reasons for the poor efficacy of immune checkpoint inhibitors in PC. Additionally, Tregs and myeloid suppressor cells drive immunosuppression in the TME of PC. Considering the unsatisfactory results of immunotherapy in the treatment of castration-resistant PC, immunotherapy strategies for PC are beginning to turn to combination regimens to enhance antitumor immune responses.

USPs are considered important immune regulators and are involved in different aspects of immune function, from innate immunity and inflammation to activation and differentiation of immune cells. USP4 depletion impairs the suppressive function of Treg cells and upregulates gene expression levels of inflammatory cytokines, such as IL-4, 5, and 13 (111). The addition of USP inhibitors to the combination therapy regimen may help to break through the current immune dilemma of PC. Multiple USPs promote PC development through different cancer-related signaling pathways. Similar to the development of inhibitors for other targets, the specificity of small molecule inhibitors remains a great challenge. The developed small-molecule inhibitors that can be used in the clinic should ensure high specificity and do no harm normal cells.

Ideally, inhibitors of USPs should act on the deubiquitination of specific substrates without altering the overall protein levels. To develop ideal inhibitors of USPs, the next step should be a more comprehensive assessment of the cellular effects of USP inhibition. Taking USP14 as an example, substrates other than desired targets of USP14 can be identified by proteomics, which is very important to improve the selectivity of the USP14 inhibitors. At present, the specific relationship between USPs and the TME in PC is still not clear enough, and there is still a long way to go before the small molecule inhibitors of USPs are successfully applied in the clinic.

## Author contributions

JG and JZ drafted the manuscript. LT and CY designed and revised the manuscript. All authors contributed to the manuscript and approved the submitted version.

## Funding

This work was supported by Zhejiang Public Welfare Technology Application Research Project (Grant No. LGF22H160038).

## Conflict of interest

The authors declare that the research was conducted in the absence of any commercial or financial relationships that could be construed as a potential conflict of interest.

## Publisher’s note

All claims expressed in this article are solely those of the authors and do not necessarily represent those of their affiliated organizations, or those of the publisher, the editors and the reviewers. Any product that may be evaluated in this article, or claim that may be made by its manufacturer, is not guaranteed or endorsed by the publisher.
